# The effect of vasopressin on the hemodynamics in CABG patients

**DOI:** 10.1186/1749-8090-8-49

**Published:** 2013-03-16

**Authors:** Hu Yimin, Liu Xiaoyu, Hu Yuping, Li Weiyan, Li Ning

**Affiliations:** 1 Department of Anesthesiology, Jinling hospital, medical school of Nanjing University, Nanjing, 210002, P.R. China; 2 Department of General Surgery, Jinling hospital, medical school of Nanjing University, No. 305, East Zhongshan Road, Nanjing, 210002, P.R. China

**Keywords:** Vasopressin, Norepinephrine, CABG, Hemodynamics

## Abstract

**Background:**

Vasopressin is widely used to treat various type of hypotension, but the effect of vasopressin on coronary artery bypass grafting surgery (CABG) patients is not clear. This study was to investigate the effect of vasopressin on the hemodynamics in CABG patients.

**Methods:**

Twenty coronary artery disease (CAD) patients were randomly divided into two groups: norepinephrine group and vasopressin group. During the anesthesia and the operation, the central venous pressure (CVP) and pulmonary capillary wedge pressure (PCWP) were maintained to 8-10cmH_2_O, and the hemocrit was maintained above 30% through lactate ringer’s mixture, artifact colloid and red blood cells. The invasive artery blood pressure (IBP) was maintained by appropriate anesthetic depth and norepinephrine or vasopressin respectively. The target IBP was 70 mmHg, and heart rate (HR) was 60 bpm. The MAP (mean artery pressure), HR, ST-T, CVP, PAP (pulmonary artery pressure), PCWP, SVR (systemic vascular resistance), PVR (pulmonary vascular resistance), CO (cardiac output), urine output, blood gas analysis, surgery duration and blood loss were monitored.

**Results:**

The MAP, HR, and ST-T were stable in either group during the operation. CVP, PCWP and SVR increased but CI deceased during the posterior descending artery (PDA) was grafted in both groups and without any significant difference between them. PAP increased during PDA was grafted in either group and there was significant difference between the two groups. PVR increased during ADA and PDA being grafted in norepinephrine group but not in vasopressin group. Metoprolol usage was 11.2 mg and 5.9 mg in norepinephrine group and vasopressin group respectively.

**Conclusion:**

Vasopressin was better than norepinephrine.to keep the hemodynamics stability of patients undergoing CABG surgery.

## Background

Coronary artery bypass grafting surgery (CABG) is a complicated surgery. The hemodynamics will be dramatically fluctuated during the operation. It is inevitable to use a series of vasoactive drugs such as norepinephrine, phenylephrine during CABG. However, there are several studies reported that hemodynamic abnormalities was corrected by using non-catecholamine drugs. Vasopressin has potential vasoconstriction effect and it could increase the afterload of the heart, therefore decreasing the cardiac output. However, the previous studies demonstrated that vasopressin could improve left ventricular function [1] and exactly increase the cardiac output and the coronary blood flow, which was ultimately due to the increase of coronary perfusion pressure [2].

Some studies have indicated that vasopressin could ameliorate hypotension effectively during some surgeries [3], especially for those patients unresponsive to norepinephrine, phenylephrine and other catecholamine vasoactive drugs. Therefore, this study was designed to investigate the effect of vasopressin on the hemodynamics during CABG surgeries.

## Methods

The study protocol followed the Declaration of Helsinki and was approved by the Ethics and Research Committee of Nanjing University (Nanjing, P.R. China). The study was performed in a prospective randomized manner after all the patients signed written informed consents. Twenty coronary artery disease (CAD) patients undergoing selective CABG surgeries were enrolled in this study.

Patients with preoperative unstable conditions such as severe arrhythmia, heart failure or cardiogenic shock, blood pressure unstability, diabetes mellitus, pulmonary disease, valvular disease, pulmonary hypertension were excluded in this study. Patients with significant pathological changes in the liver and renal function and needing emergency cardiopulmonary bypass should also be excluded.

All the patients were monitored with ECG, IBP, SpO_2_ after they entered the operating rooms. Anesthesia induction program: midazolam 0.05 mg/kg, etomidate 0.1-0.2 mg/kg, vecuronium bromide 0.15 mg/kg, fentanyl 5-10 ug/kg. Then central venous and Swan-ganz catheters were placed. Anesthesia maintenance program: Propofol 6-10 mg/kg/h, atracurium 5-10 ug/kg/min, fentanyl intermittently. The total fentanyl dosage was controlled to 30-50 ug/kg. The anesthesiologists, surgeons, nurses and the statistician were blinded to the protocol. Patients were randomly divided into two groups by a computer program. Ten patients were administered norepinephrine and the others were administered vasopressin in the presence of hypotension during the operation. Using Ringer's lactate, artificial colloidal and red blood cells to maintain CVP and PCWP, to keep HCT not less than 30%, and to obtain IBP 70-90 mmHg,HR 55-65 bpm. IBP, HR, ST-T, CVP, PAP, SVR, PVR, CO, urine output, arterial blood gas analysis, the operation duration, the amount of bleeding, drug dosage, beta block usage were recorded.

### Statistical analysis

The data were analyzed with the software SPSS 13.0. The quantitative data were expressed as mean ± SD, and repeated measures analysis was used for hemodynamic variables. Also, unpaired Student t for difference in artery blood gas analysis and other quantitative parameters between the two groups. The qualitative data were compared with chi-square analysis. Fisher’s exact test was used when the minimum expected count was less than five. *P* < 0.05 was considered to be significant.

## Results

**Figure 1:**
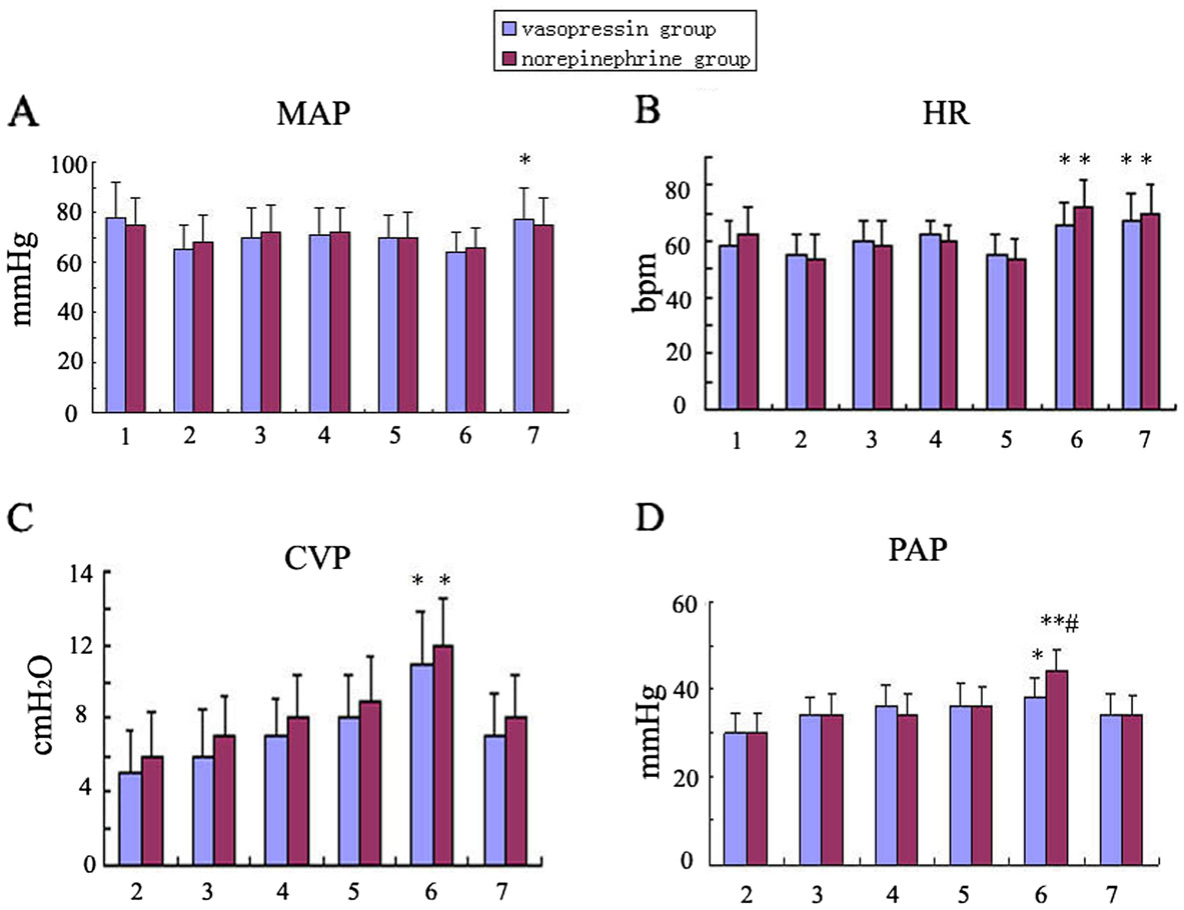
**A: MAP changes in the two groups. B**: HR changes in the two groups. **C**: CVP changes in the two groups. **D**: PAP changes in the two groups. MAP: mean artery pressure, HR: heart rate, CVP: central venous pressure, PAP: pulmonary artery pressure. *P < 0.05, **P < 0.01 VS baseline. #P < 0.05,##P < 0.01 within the groups.

**Figure 2:**
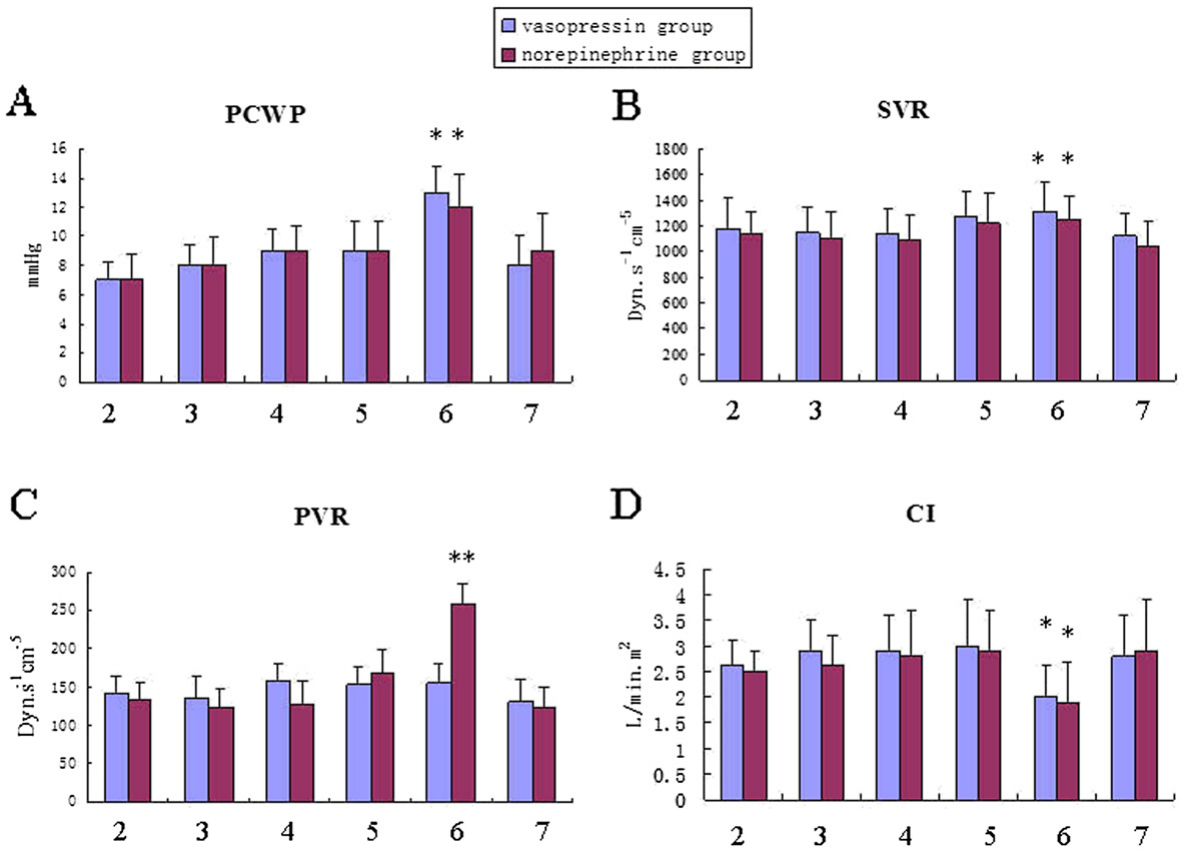
**A: PCWP changes in the two groups. B**: SVR changes in the two groups. **C**: PVR changes in the two groups. **D**: CI changes in the two groups. PCWP: pulmonary capillary wedge pressure, SVR: systemic vascular resistance, PVR: pulmonary vascular resistance, CI: cardiac index. *P < 0.05, **P < 0.01 VS baseline. #P < 0.05,##P < 0.01 within the groups.

There was no significant difference in the age, gender, BMI, heart function, ASA physical status, baseline MAP and HR between the two groups. All patients had three coronary artery lesions and received three grafts. There was no significant difference in MAP after induction of anesthesia and during surgery between the two groups. MAP is higher than that after anesthesia induction but close to the baseline value at the end of surgery. CVP, PCWP, SVR and CI increased significantly during PDA grafting was performed and there was no difference between groups. PAP and PVR also increased during PDA grafting was performed and there was difference between groups. (Figures 1 and 2).

Acid–base balance and blood gas analysis: Hemoglobin concentration decreased significantly during ADA and PDA was performed and at the end of surgery, there was significant difference when compared with that after anesthesia induction (*P* < 0.05), however, no significant difference was found between the two groups (Table 1).

**Table 1:** Artery blood gas analysis during the two groups

	**PH**	**PO2**	**PCO2**	**BE**	**HB**	**Na**	**K**	**iCa**
vasopressin group								
Baseline	7.36 ± 0.09	479 ± 33	42 ± 2.2	−1 ± 0.9	13.8 ± 3.5	139 ± 9	3.7 ± 0.2	1.15 ± 0.15
After induction of anesthesia	7.33 ± 0.11	509 ± 35	44 ± 2.5	0 ± 0.4	13.1 ± 3.3	133 ± 8	3.6 ± 0.5	1.11 ± 0.11
5 min after surgical incision	7.39 ± 0.11	516 ± 21	45 ± 1.9	−2 ± 0.6	12.9 ± 2.5	135 ± 9	3.3 ± 0.3	1.15 ± 0.21
5 min after Sternotomy	7.40 ± 0.12	467 ± 22	42 ± 2.6	−1 ± 0.9	10.5 ± 2.2*	136 ± 8	3.8 ± 0.5	1.06 ± 0.22
During ADA grafting	7.30 ± 0.08	444 ± 20	39 ± 3.2	−2.5 ± 1.1	11.1 ± 3.7*	139 ± 7	3.9 ± 0.3	1.08 ± 0.30
During PDA grafting	7.41 ± 0.07	489 ± 31	44 ± 3.2	−1 ± 0.2	11.0 ± 2.3*	138 ± 7	4.2 ± 0.4	1.22 ± 0.11
The end of surgery	7.39 ± 0.07	501 ± 33	43 ± 2.6	−2 ± 0.3	12.9 ± 2.6	142 ± 6	3.9 ± 0.2	1.25 ± 0.09
norepinephrine group								
Baseline	7.33 ± 0.11	523 ± 30	45 ± 2.2	0 ± 0.6	12.1 ± 2.8	143 ± 6	3.6 ± 0.2	1.21 ± 0.08
After induction of anesthesia	7.36 ± 0.10	498 ± 28	45 ± 2.7	−2 ± 0.9	11.9 ± 1.9	139 ± 5	3.8 ± 0.4	1.15 ± 0.12
5 min after surgical incision	7.41 ± 0.12	489 ± 26	43 ± 3.2	−2 ± 1.0	10.0 ± 1.5*	135 ± 7	3.9 ± 0.3	1.16 ± 0.11
5 min after Sternotomy	7.40 ± 0.13	465 ± 27	41 ± 3.6	−1.5 ± 1.0	11.5 ± 1.6*	137 ± 8	4.2 ± 0.3	1.18 ± 0.12
During ADA grafting	7.41 ± 0.12	489 ± 26	43 ± 3.2	−2 ± 1.0	10.0 ± 1.5*	135 ± 7	3.9 ± 0.3	1.16 ± 0.11
During PDA grafting	7.40 ± 0.13	465 ± 27	41 ± 3.6	−1.5 ± 1.0	11.5 ± 1.6*	137 ± 8	4.2 ± 0.3	1.18 ± 0.12
The end of surgery	7.42 ± 0.09	500 ± 22	45 ± 3.2	0 ± 0.5	11.0 ± 1.8*	139 ± 9	4.4 ± 0.5	1.24 ± 0.13

*P < 0.05 VS baseline.

There was no obvious abnormality in ST-T segment, operation duration, bleeding volume and urine output in the two groups patients during surgery. Total vasopressin usage was 3.6 IU in vasopressin group and total norepinephrine usage was 0.48 mg in norepinephrine group. Metoprolol usage was 5.9 mg in vasopressin group and 11.2 mg in norepinephrine group (*P* < 0.01) (Table 2).

**Table 2:** Other parameters during the two groups

	**Operation time (h)**	**The amount of bleeding (ml)**	**Urine output (ml)**	**Drug dosage**	**Metoprolol dosage mg**
vasopressin group	4.6 ± 0.6	680 ± 110	1250 ± 202	0.4 ± 0.11 mg	11.2 ± 3.4
norepinephrine group	4.4 ± 0.7	660 ± 99	1365 ± 189	3.6 ± 1.2 IU	5.9 ± 2.0##

## Discussion and conclusion

The hemodynamics showed dramatic fluctuation in CABG patients during the anesthesia and surgery, which is probably due to its pathophysiological characteristics and operative factors. In this study, these patients were poorly tolerated in the presence of both anesthesia and surgery. Anesthesiologists should maintain the stability of hemanynamics, so some vasoactive drugs such as phenylephrine, norepinephrine, and other catecholamine drugs are inevitably used.

There are three intrinsic vasopressor systems, ie, sympathetic nervous system, renin- angiotensin system and vasopressin system. Mounting studies have indicated that vasopressin was much more potent in cardiopulmonary resuscitation than catecholamines [4]. But for CABG patients, it was unclear which vasopressor was more potent and with less side effects.

Our study confirmed that the vasopressin is similar to the traditional catecholamines to maintain the stability of hemodynamics, and even superior to the catecholamines in some aspects, such as pulmonary artery pressure, metoprololuse usage. Catecholamine hormones could increase blood pressure, while inevitablely increase pulmonary vascular resistance, thereby leading to increase of pulmonary artery pressure. Pulmonary hypertension, as we know, a very dangerous pathophysiological change, deteriorate hemodynamic and respiratory function, meanwhile induce the failure of the entire surgery.

No evidence showed that using of low doses of vasopressin (0.04 U/min) increased PVR or PAP. On the contrary, Basic study showed that vasopressin could decrease PAP [5]. The results of this study suggested that no pulmonary artery pressure elevation after administration of vasopressin, instead, pulmonary artery pressure is relatively stable in vasopressin group perhaps due to the better-maintained systemic arterial pressure and less norepinephrine usage.

Patients undergoing CABG surgery are needed to maintain a slower heart rate to reduce myocardial oxygen consumption and facilitate the surgical procedure. Although CABG patients will have a slower heart rate during the anesthesia and operation because of pre-operative β-blockers and anesthetic agents, but the heart rate still inevitable increase during the surgery and anesthesiologist need to use the additional beta blockers. In this study, vasopressin group patients need less beta blockers but not in norepinephrine group patients. The reasons may be norepinephrine has positive inotropic and positive frequency effect and pulmonary pressure is relatively unstable.

Vasopressin, also known as antidiuretic hormone can reduce urine output. But this study did not indicate an obvious decreament in the urine output after treatment of vasopressin. Enough arterial pressure and cardiac output can assure enough kidney perfusion and result in maintained urine output. Antidiuretic hormone can enhance the kidney collecting duct re-absorption of H2O, leading to dilutional hyponatremia. Serum sodium and other electrolytes should be monitored during prolonged use of vasopressin.

In addition, it was generally believed that high blood pressure can lead to increased surgical drainage. But in this study vasopressin increased blood pressure without increasing the amount of bleeding volume. Some study demonstrated that the anti-diuretic hormone receptor has three subtypes, V1, V2 and V3 receptors. V2 receptor exciting can increase the concentration of plasma coagulation factor VII and vWF. Consequently, researchers have already proposed: for those patients with reduced plasma level of factor VIII and vWF such as hemophilia A and type I von Willebrand disease, anti-diuretic hormone should be administered during perioperative time [6].

Vasopressin can also cause a transient elevation in blood glucose and the mechanism is related to the liver glycogen catabolism by V1 receptor. We recommended that patients’ blood glucose should be monitored for prolonged use of vasopressin and those with diabetic mellitus [7].

In conclusion, vasopressin may be another choice to deal with hypotension during CABG surgeries.

## Competing interests

The authors declare that they have no competing interests.

## Authors’ contribution

Hu Yimin did the majority of the research and wrote the entire article. Liu Xiaoyu helped Hu Yimin to complete the clinical research. Hu Yuping did the statistic. Li Weiyan directed the research. Li Ning is the correspondence author and responsible to the research. All authors read and approved the final manuscript.
